# Association of non-high-density lipoprotein to high-density lipoprotein ratio (NHHR) with prognosis in cancer survivors: a population-based study in the United States

**DOI:** 10.3389/fnut.2024.1430835

**Published:** 2024-09-04

**Authors:** Wenxia Xie, Huizhuo Liu, Qiaoxin Lin, Liyou Lian, Bin Liang

**Affiliations:** ^1^Department of Oncology, The First Affiliated Hospital of Wenzhou Medical University, Wenzhou, China; ^2^Department of Internal Medicine, The First Affiliated Hospital of Wenzhou Medical University, Wenzhou, China

**Keywords:** non-HDL-C/HDL-C (NHHR), cancer survivors, all-cause mortality, cardiac-specific mortality, cancer-specific mortality, NHANES

## Abstract

**Background:**

Patients with cancer frequently exhibit alterations in serum lipid profiles associated with chemotherapy. It has been reported that lipid distribution in cancer correlates with tumor progression. However, the prognostic value of serum lipid biomarkers in cancer survivors remains a subject of debate. We aim to explore the relationship between non-high-density lipoprotein to high-density lipoprotein ratio (NHHR) and the prognosis of cancer survivors.

**Methods:**

In this study, we analyzed cancer survivor data from the National Health and Nutrition Examination Survey (NHANES) from 1999–2000 to 2017–2018. The study included prospective cohorts that included total cholesterol (TC) and high-density lipoprotein cholesterol (HDL-C) levels as well as mortality data. Weighted multivariate cox regression models, competing risk models and restricted cubic spline (RCS) models were applied to investigate the association between NHHR and cancer survival. Subgroup and sensitivity analyses were performed to test the robustness of the results.

**Results:**

This study involved 4,177 participants, representing about 19.6 million U.S. adults. After adjustment for various factors, the lower NHHR group (≤1.64) had a 31% (HR 1.31; 95% CI [1.11,1.54], *p* = 0.001) higher risk of death from any cause compared to the higher NHHR group. The link between NHHR and mortality remained stable across most subgroups, with notable interactions for smoking (*p* = 0.006) and diabetes status (*p* = 0.046). A J-shaped pattern was observed between NHHR and all-cause mortality, significantly among obesity-related cancer survivors (overall association test *p*-value = 0.0068, non-linear association test *p*-value = 0.0016). However, a non-significant negative correlation was observed for cancer-specific mortality (overall association test *p*-value = 0.48, non-linear association test *p*-value = 0.66). Considering the competitive risk of heart disease and cancer-specific mortality, there is no difference between the high and low NHHR groups, while the low NHHR group showed an increased risk of non-specific causes of death (*p* < 0.001).

**Conclusion:**

The results of this study suggest that NHHR is an important indicator that is strongly associated with all-cause mortality in cancer survivors, and that this relationship may be influenced by the interaction of diabetes and smoking status. This finding may provide important information for future research and patient management.

## Introduction

The prevalence of cancer survivors has markedly augmented due to the senescence of the populace coupled with advancements in the realms of early detection, diagnostic procedures, and treatment modalities, with an estimated 69% of these individuals achieving a survival span extending beyond 5 years (1). It is projected that the population of cancer survivors within the United States will escalate to 26 million by the year 2040 (2). Consequently, there has been a synchronous escalation in the incidence of cancer survivors with comorbid conditions. Relative to the control cohort, a greater incidence of poly-pathology, defined as the presence of four or more concurrent diseases, was reported among cancer survivors (57% as opposed to 38%) (3). As cancer survival rates improve, competing causes of mortality and morbidity become more important among cancer survivors. Epidemiologic data suggest that cancer survivors have an increased risk of cardiovascular disease (4, 5). This phenomenon might be attributable to an intrinsic interconnection among these conditions; overlapping risk determinants encompass hypertension, hyperlipidemia, diabetes mellitus, adiposity, tobacco use, dietary habits, physical activity levels, along with various social health determinants. Additionally, shared pathophysiological processes such as persistent inflammation, heightened oxidative stress, metabolic imbalance, compromised immune functionality, ambiguous clonal hematopoiesis, microbiome imbalance, hormonal influences, and enhanced cellular aging could also play a contributory role (6, 7). Notable advancements in the creation of novel cancer treatments have markedly revolutionized the management of numerous malignancies. However, the enduring complications associated with cancer and its therapeutic interventions may also elevate the risk of cardiovascular disease (CVD) (8). Currently, the range of cardiotoxic effects encompasses not merely those related to traditional cancer treatments (such as anthracyclines, trastuzumab, or radiotherapy) but extends to cardiac impairment resulting from myocarditis induced by immune checkpoint inhibitors and cytokine release syndrome caused by chimeric antigen receptor T-cell therapy (9). In addition to prolongation of the widened QT interval, the incidence of arrhythmias associated with inflammation (e.g., atrial fibrillation) may also increase (10, 11). Meanwhile, CVD is becoming the predominant cause of mortality for some cancer survivors (5, 12). In some cancers, the current risk of non-cancer deaths now exceeds the risk of cancer deaths (13). Hence, it becomes imperative to consider cardiovascular disease when evaluating treatment methods and cancer prognosis to improve the care delivered to patients suffering from both cardiovascular disease and cancer (14).

Plasma lipoprotein cholesterol (PLC) is commonly used as an indicator of cardiovascular disease risk assessment. High-density lipoprotein cholesterol (HDL-C) is often thought of as the “good” cholesterol, which transports cholesterol in the blood and escorts it to the liver for processing and excretion (15). Whereas non-HDL cholesterol (non-HDL-C), which includes low-density lipoprotein cholesterol (LDL-C) and other lipoproteins, is thought to contribute to atherosclerosis formation and increase the risk of CVD (16). Relative to the lowest quartile, higher quartiles of TC, LDL-C, and non-HDL-C all correlated with a heightened risk of atherosclerotic cardiovascular disease (ASCVD); conversely, these factors were inversely associated with the risk of cancer (17). Previous work has found that cancer patients often show abnormal lipid profiles (18). And new evidence suggests that lipids measured during cancer treatment may serve as biomarkers for monitoring cardiovascular health in cancer survivors and are associated with cancer development (19–22) and CVD and cardiovascular-specific mortality (17, 23). In addition, more and more studies show the correlation between PLC and malignant tumor, including the important role of tumor cell proliferation, angiogenesis, immunoregulation and other processes. However, there is no definite relationship between PLC and cancer mortality (24, 25).

Despite the prevalent occurrence of cardiovascular diseases among cancer survivors, consensus on the best practices for monitoring and assessing cardiovascular health during cancer treatment has yet to be established. The current challenge lies in the absence of a standardized and systematic approach for quantifying cardiovascular health indicators that are associated with the long-term prognosis of cancer survivors. In this context, understanding the characteristics and physiological functions of non-HDL-C and HDL-C becomes particularly crucial (26). Nonetheless, the associations between non-HDL-C and HDL-C levels and all-cause mortality, cardiac-specific mortality, and cancer-specific mortality among cancer survivors remain unclear. This study utilized data from the National Health and Nutrition Examination Survey (NHANES) database to explore the potential links between lipid metabolism and prognosis among cancer survivors. It is imperative to emphasize that the roles of HDL and non-HDL-C should not be analyzed in isolation; they should be considered as part of the extensive network of lipid metabolism within the body. Therefore, this study aims to reveal the role of the non-HDL-C/HDL-C ratio (NHHR) in the prognosis of cancer patients, thus offering a new perspective on monitoring cardiovascular health.

## Methods

### Research participants

The dataset for this study was derived from the NHANES database, which has been conducted over 10 continuous cycles from 1999–2000 to 2017–2018. NHANES is administered and managed by the National Center for Health Statistics (NCHS), under the auspices of the Centers for Disease Control and Prevention (CDC). This nationally representative cohort study utilizes a complex, stratified, multistage probability design to survey non-institutionalized civilians in the United States at mobile examination centers. Survey components include demographic data, physical examinations, laboratory tests for biomarkers, dietary assessments, and psychophysiological questionnaires, all of which facilitate the evaluation of the relationship between nutritional status and disease.

**Figure 1:**
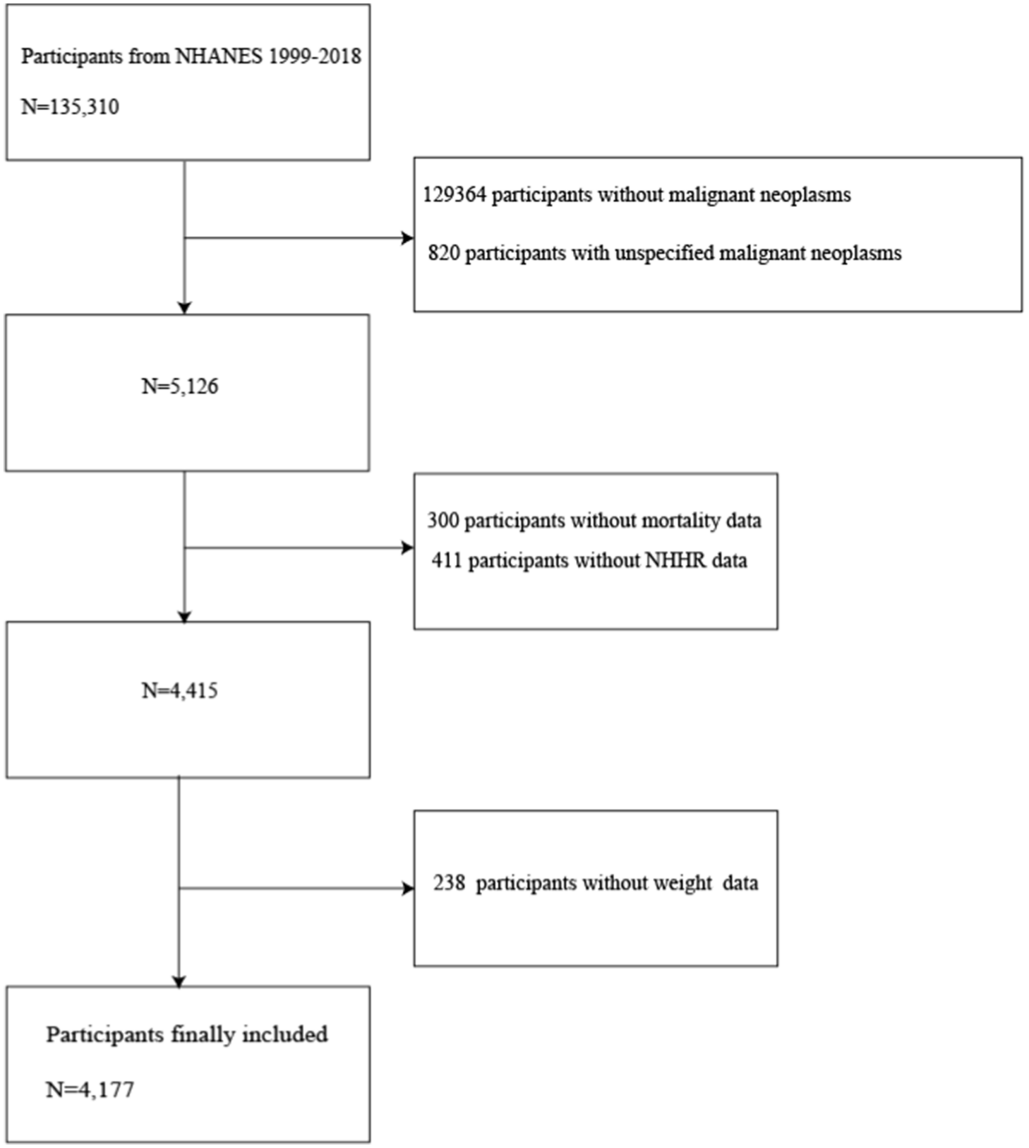
Flowchart of participant selection. NHANES, National Health and Nutrition Examination Survey; NHHR, non-high-density lipoprotein cholesterol to high-density lipoprotein cholesterol ratio.

This research was rigorously conducted in accordance with the STROBE (Strengthening the Reporting of Observational Studies in Epidemiology) guidelines. Among 135,310 participants, cancer survivors who were 20 years of age or older (*N* = 5,126) were chosen for subsequent follow-up. Participants with missing data were excluded, including those with missing follow-up information (*N* = 300), NHHR (*N* = 411), and missing weights (*N* = 238). As shown in Figure 1, a total of 4,177 samples were ultimately available for analysis. To address the issue of missing variables and ensure the statistical power of accurately describing the target sample during the modeling phase, this study employed multiple imputation to handle missing data (27, 28). The imputation model included a wide range of variables, encompassing sex, age, marital status, race, Poverty Income Ratio (PIR), Body Mass Index (BMI), Healthy Eating Index-2015 (HEI2015), Leisure time physical activity (LTPA), smoking status, drinking status, diabetes, hypertension, renal failure, and CVD. The analysis of missing data followed the assumption of Missing at Random (MAR) to ensure the effectiveness of the imputation process (28). The timeline for data analysis was from March 2024 to April 2024. Ethical approval for NHANES was granted by the National Center for Health Statistics Ethical Review Board, with the following approval numbers: Protocol #2018-01, Protocol #2011–17 Continuation, Protocol#2011-17, Protocol#2005–06 Continuation, Protocol#2005–06 and Protocol#98-12. Given that this research is based on already published and nationally representative de-identified datasets that do not include personal identity information, patient informed consent was not required. Further information can be acquired from the following website: NCHS Ethical Review Committee Approval.^1^ Research data is available at the website: https://www.cdc.gov/nchs/nhanes/index.htm.

### Diagnosis of cancer

Data on cancer diagnoses, encompassing the cancer varieties, were amassed through direct interviews. The subjects were probed with the question, “Has a healthcare provider ever informed you that you had cancer or a malignancy of any sort?” Individuals confirming such a diagnosis were categorized as cancer survivors and were further inquired about the specific cancer variety they suffered from. The exclusion criteria ruled out participants with a history of more than three cancer types, as well as those who were unavailable for subsequent follow-up, those who answered with uncertainty, or those who declined to provide a response.

### Ascertainment of mortality

Mortality information until December 31, 2019, was acquired from the NCHS, which was matched with the records from the National Death Index. To ascertain the primary causes of demise, ICD-10 was employed for coding. Deaths attributed to cardiac ailments were defined under the categories of heart-related diseases (codes I00-I09, I11, I13, and I20-I51) and those due to cancer were specified as those resulting from malignancies (codes C00-C97). The follow-up period was measured in months, commencing from the examination until the event of death, or up to December 31, 2019, for those who did not succumb (29).

### Selection of research variables

In the assessment of exposure, NHHR is utilized as an independent variable. Following methodologies established in preceding research, the NHHR was quantified by the ratio of Non-HDL-C to HDL-C (30). The deduction of HDL-C from the TC yielded the non-HDL-C, a parameter ascertained through the lipid profile analysis of individuals in a fasting state. The concentration of TC and the assessment of HDL-C were carried out using enzymatic assays by automated biochemical analyzers, specifically the Roche Cobas 6,000 and the Roche Modular P systems were employed for the determination of TC levels in the studies’ analytical phase.

The incorporation of covariates in this study is rooted in a thorough review of past literature, clinical expertise, and the accomplishment of statistical significance. During household interviews, demographic characteristics such as age, gender, race, marital status, education level, and PIR were collected. Physical measurements were taken by trained technicians, including height, weight, and the average of three consecutive blood pressure readings obtained while participants were seated and at rest for 5 min. Hypertension is defined as: (1) a self-reported history of hypertension; (2) current use of antihypertensive medication; or (3) an average systolic blood pressure of ≥140 mmHg and/or an average diastolic blood pressure of ≥90 mmHg. Individuals who met one or more of the following criteria were considered to have diabetes: (1) fasting plasma glucose ≥7.0 mmol/L; (2) glycohemoglobin (HbA1c) ≥6.5%; (3) use of diabetic medications or insulin; and (4) self-reported diabetes.

Lifestyle factors were captured through self-reported responses, including smoking, alcohol consumption, and physical activity. “Never” was designated for those who smoked less than 100 cigarettes in their lifetime; individuals who had smoked ≥100 cigarettes in their lifetime but presently do not smoke were considered “former”; those who had smoked ≥100 cigarettes in their lifetime and currently smoke every day or some days were categorized as “current.” Participants were classified as “never,” “former,” or “current” drinkers based on the frequency of alcohol consumption over the past year: “never” (lifetime <12 drinks), “former” (no drinks in the past year but ≥12 drinks annually or in the lifetime), and “current” (drank in the past year and ≥12 drinks annually or in the lifetime). LTPA was divided into three categories based on Metabolic Equivalent Task (MET) scores: 0 min/week*MET, <800 min/week*MET, and ≥800 min/week*MET. Additionally, dietary data obtained from 24-h dietary recalls were used to assess energy intake.

Thirteen types of obesity-related cancers were grouped: esophageal adenocarcinoma, gastric cardia cancer, colorectal cancer, liver cancer, gallbladder cancer, pancreatic cancer, uterine cancer, ovarian cancer, breast cancer, renal cell carcinoma, thyroid cancer, meningioma, and multiple myeloma, which have been robustly evidenced in literature as associated with obesity (31).

### Statistical analysis

Statistical analyses were conducted using R software (Version 4.2.3, The R Foundation). Bilateral *p*-values less than 0.05 were considered to indicate statistical significance. To ensure result accuracy, data were weighted according to NHANES analytical guidelines, addressing the inherent complex sampling design of the dataset. We chose WTDRD1, which involves the smallest subset of variables. The weight of 1999–2002 (2 cycles) is 2/10*WTDR4YR; the weight of 2003–2018 (8 cycles) is 1/10*WTDRD1. Categorical variables were expressed as weighted frequencies and corresponding percentages. To assess differences between groups, the Rao-Scott chi-squared test was utilized. Continuous variables were described as weighted means (SD). Weighted *t*-tests were employed for intergroup comparisons of continuous variables.

The optimal NHHR cutoff point corresponding to the most significant association with survival outcomes was obtained using maximally selected rank statistics based on the ‘maxstat’ package,^2^ which were then used to separate participants into high-and low-NHHR groups. Kaplan–Meier survival curves, with the log-rank test, were employed to evaluate the survival probabilities of cancer patients at different levels of NHHR. The relationship between NHHR and all-cause mortality was evaluated using the survey-weighted multivariable Cox regression model. Three models were constructed to adjust for possible confounders. Model 1 was adjusted for NHHR group, age, gender, race, education level, PIR, and marital status. Model 2 was adjusted for NHHR group, age, gender, race, education level, PIR, marital status, BMI, HEI-2015, LTPA, smoking status, and alcohol status. Model 3 was additionally adjusted for NHHR group, age, gender, race, education level, PIR, marital status, BMI, HEI-2015, LTPA, smoking status, alcohol status, diabetes, hypertension, cardiovascular disease, obesity-related tumors, and renal failure. The association of NHHR values with all-cause mortality was analyzed using subgroups based on demographic characteristics, lifestyle habits, and comorbidities, and their interactions were explored. Time-dependent Receiver Operating Characteristic (ROC) curve analyses were conducted on the adjusted, weighted multivariate Cox regression models to evaluate the precision of the model in predicting survival outcomes. Potential non-linear associations between NHHR and specific and non-specific mortality risks were visualized using restricted cubic spline (RCS) with three knots. Finally, we defined specific deaths (cardiac deaths and cancer deaths) and deaths from other causes as mutually exclusive events, and assessed the relationship between NHHR group and both specific and non-specific mortality through a competing risk model. We used Fine-Gray subdistribution hazard models that accounted for the competing risk of death and expressed associations as subdistribution hazard ratios.

## Results

### Characteristics of the study population

**Figure 2:**
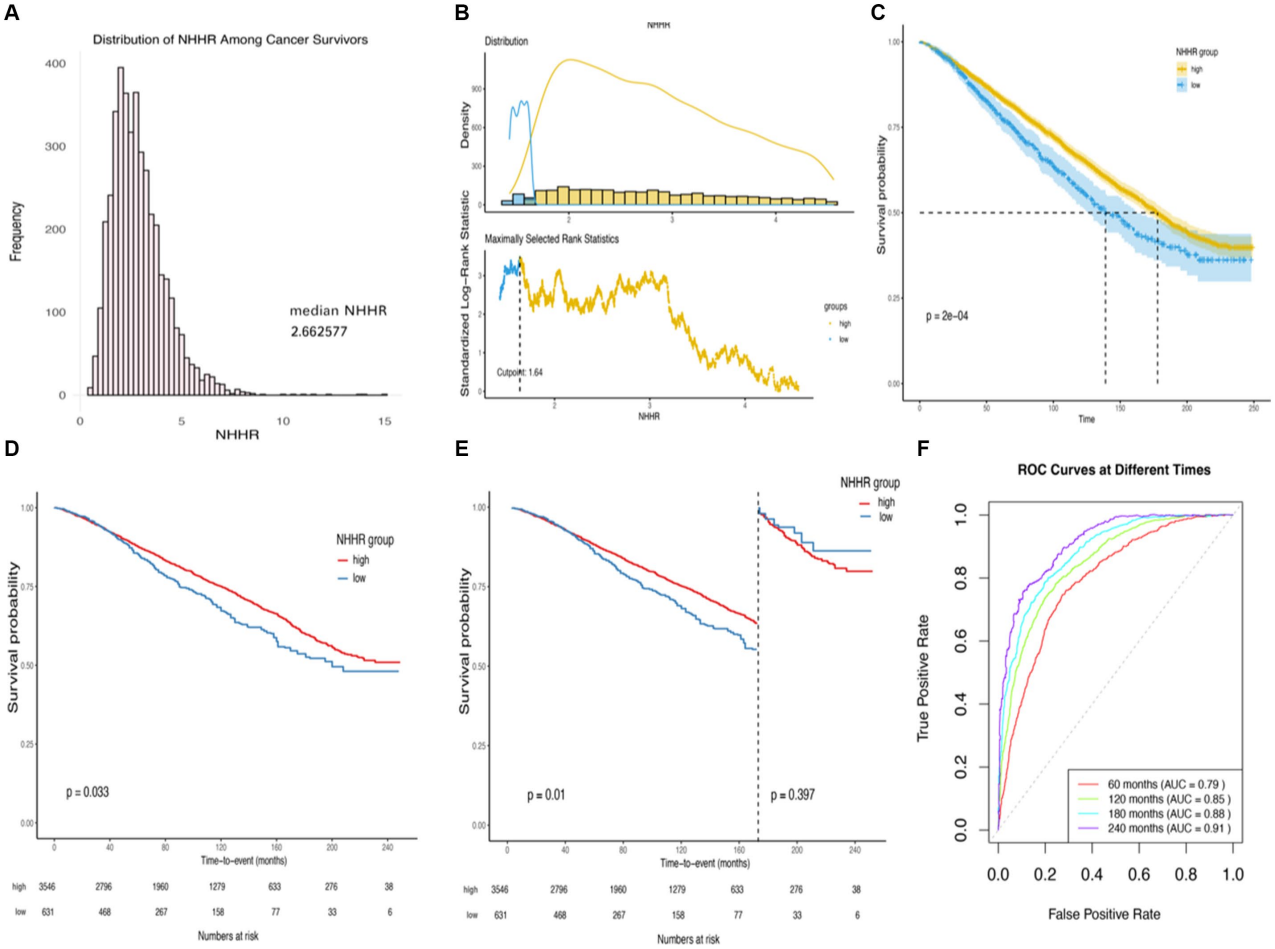
Kaplan–Meier survival rates illustrating mortality among US adult cancer patients categorized by different NHHR groups: **(A)** Distribution of NHHR among all cancer survivors. **(B)** The cutoff is calculated using the maximally selected ranking statistic based on the “maxstat” package. **(C)** Kaplan–Meier Curves for survival in cancer survivors with High NHHR (>1.64) and Low (≤1.64) NHHR Values. **(D)** Kaplan–Meier curves for survival in cancer survivors with high NHHR (>1.64) and low (≤1.64) NHHR values, weighted data. **(E)** Kaplan–Meier curves for survival in cancer survivors with high NHHR (>1.64) and low (≤1.64) NHHR values, weighted data, cut.landmark value of 170. **(F)** Time-dependent ROC curves for the prediction of all-cause mortality by weighted multifactorial cox regression models. Statistical adjustments were made for NHHR group, age, gender, race, education level, income-to-poverty ratio, marital status, BMI, HEI2015, LTPA, smoking status, alcohol status, diabetes, hypertension, cardiovascular disease, obesity-related tumors, and renal failure.

Our cohort consisted of 4,177 participants, representing an estimated 19,617,771 adults in the United States based on weighted estimation. The NHHR is normally distributed among cancer survivors, as illustrated in Figure 2A. Utilizing the optimal cutoff value of 1.64 for NHHR, participants were divided into high NHHR (NHHR >1.64, *n* = 3,546) and low NHHR (NHHR ≤1.64, *n* = 631) groups (Figures 2B,C). Baseline characteristics of the different NHHR level groups are detailed in Table 1.

**Table 1:** Characteristics of the study population ascertained by NHANES from 1999 to 2018.

Variable	Higher NHHR	Lower NHHR	*p*-value
Participants	16746772.9	2870998.36	
Age, Mean (SD), years	62.20 (14.38)	64.34 (15.17)	0.009
**Gender, *n* (%)**			
Female	9169368.1 (54.8)	1999316.8 (69.6)	<0.001
Male	7577404.8 (45.2)	871681.5 (30.4)	
**Race and ethnicity, *n* (%)**			
Mexican American	414455.4 (2.5)	42221.5 (1.5)	0.012
Non-Hispanic Black	786785.0 (4.7)	211140.1 (7.4)	
Non-Hispanic White	14611330.9 (87.2)	2470815.2 (86.1)	
Other Hispanic	389527.4 (2.3)	29305.0 (1.0)	
Other Race	544674.3 (3.3)	117516.5 (4.1)	
**Educational attainment, *n* (%)**			
<High school	2447301.1 (14.6)	375639.0 (13.1)	0.092
>High school	10558308.9 (63.0)	1959988.5 (68.3)	
Completed high school	3741162.9 (22.3)	535370.8 (18.6)	
Family poverty income ratio, Mean (SD)	3.25 (2.08)	3.47 (1.56)	0.029
**Marital status, *n* (%)**			
Married/Living with partner	11248919.4 (67.2)	1780586.5 (62.0)	0.061
Widowed/Divorced/Separated/Never married	5497853.5 (32.8)	1090411.9 (38.0)	
Body mass index, Mean (SD), kg/m2	29.29 (6.31)	25.50 (6.13)	<0.001
HEI2015 (Mean, SD)	52.37 (13.52)	56.07 (14.51)	<0.001
Leisure-time physical activity (Mean, SD)	828.62 (1852.63)	1072.72 (1658.70)	0.006
**Smoking status, *n* (%)**			
Current smoker	2789603.8 (16.7)	387382.9 (13.5)	0.224
Former smoker	6426091.0 (38.4)	1169107.8 (40.7)	
Never smoker	7531078.2 (45.0)	1314507.7 (45.8)	
**Alcohol status, *n* (%)**			
Current drinker	11032517.0 (65.9)	2034112.7 (70.9)	0.145
Former drinker	3917925.0 (23.4)	555146.6 (19.3)	
Never drinker	1796330.9 (10.7)	281739.1 (9.8)	
**Hypertension, *n* (%)**			
NO	6810986.3 (40.7)	1285038.9 (44.8)	0.17
YES	9935786.6 (59.3)	1585959.5 (55.2)	
**Diabetes, *n* (%)**			
NO	13326527.0 (79.6)	2455764.6 (85.5)	0.01
YES	3420245.9 (20.4)	415233.8 (14.5)	
**Cardiovascular disease, *n* (%)**			
NO	13526188.1 (80.8)	2252787.9 (78.5)	0.31
YES	3220584.8 (19.2)	618210.4 (21.5)	
**Renal failure, *n* (%)**			
NO	15871441.3 (94.8)	2688024.6 (93.6)	0.337
YES	875331.6 (5.2)	182973.8 (6.4)	
**Obesity-related tumors, *n* (%)**			
NO	11719847.8 (70.0)	1923305.7 (67.0)	0.273
YES	5026925.1 (30.0)	947692.7 (33.0)	

Compared to the high NHHR group, the low NHHR group exhibited significantly different demographic characteristics. Specifically, individuals in the low NHHR group were older, had a higher PIR, scored higher HEI2015, and had higher percentages of women and Non-Hispanic Black. Additionally, the low NHHR group spent more time participating in LTPA, as well as having a lower BMI and lower prevalence of diabetes mellitus (Table 1).

### Association of NHHR groups with all-cause mortality in cancer survivors

In the stratified analysis of adult cancer patients in the United States, a further exploration of survival among groups stratified by different levels of NHHR was performed using weighted Kaplan–Meier survival curves (Figure 2C). Compared to the patient cohort with higher NHHR, the group with lower NHHR demonstrated a significantly higher all-cause mortality rate, a difference that was statistically significant (Log-rank test *p*-value = 0.033). When analyzing survival time with a cut-off at 170 months, the association between NHHR and all-cause mortality became even more pronounced (*p*-value = 0.01) (Figures 2D,E).

**Figure 3:**
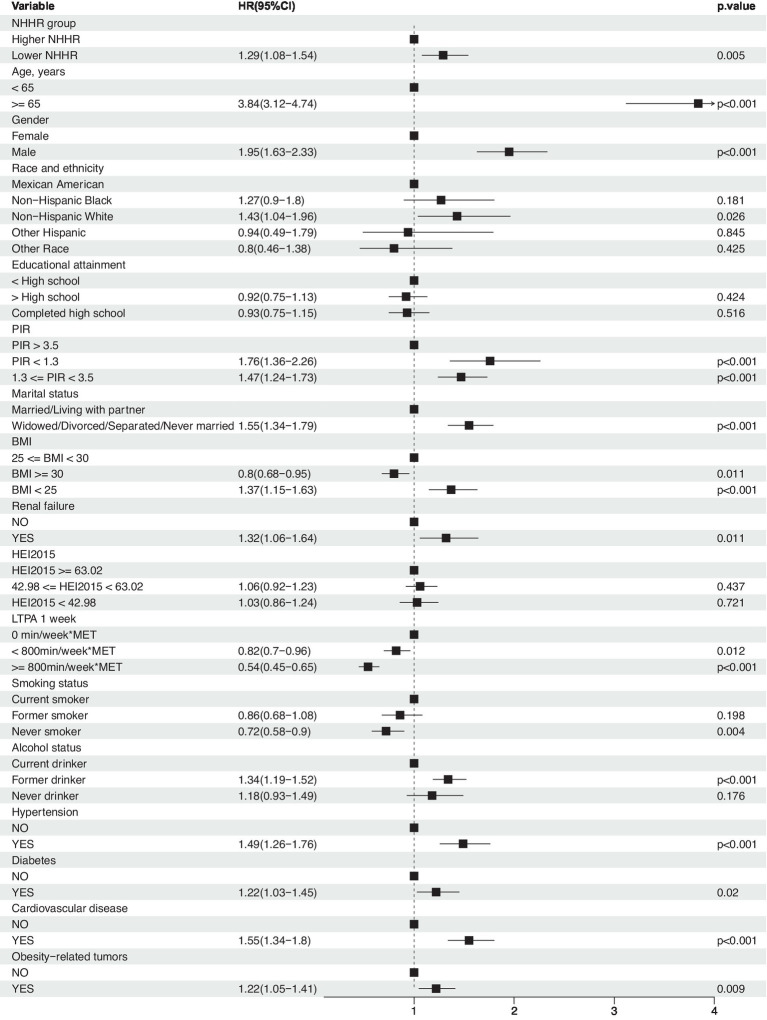
Association between NHHR and risk of all-cause mortality in cancer survivors. Weighted cox proportional hazards models adjusted for age, gender, race, education level, PIR, marital status, BMI, HEI2015, LTPA, smoking status, alcohol status, diabetes, hypertension, cardiovascular disease, obesity-related tumors, and renal failure.

Moreover, weighted multivariable Cox regression analysis indicated that, in the crude model without multivariable adjustment, the low NHHR group exhibited a significant increase in the risk of all-cause mortality compared to the high NHHR group [Hazard Ratio (HR) = 1.23, 95% Confidence Interval (CI) = 1.02 to 1.49, *p*-value = 0.029] (Table 2). After multivariable adjustments, the risk of all-cause mortality in the low NHHR group increased by 29, 32, and 31% across three models, respectively, compared to the high NHHR group (Table 2). Other survival-related factors include age, gender, PIR, marital status, BMI, renal failure, LTPA, smoking status, alcohol status, hypertension, diabetes, CVD, obesity-related tumors, etc. (Figure 3). The Concordance value of Model 3 was 0.803. Time-based ROC analysis was also conducted to assess the model’s predictive value for all-cause mortality among cancer survivors. The results showed that the Area Under the Curve (AUC) for the model’s predictive values for all-cause mortality at 5, 10, 15, and 20 years was 0.79, 0.85, 0.88, and 0.91, respectively (Figure 2F). These findings suggest that the model appears to have efficient predictive value for all-cause mortality in both the short and long term. In an analysis of obesity-related tumors, a significant association between lower NHHR and higher all-cause mortality was also observed (Table 2).

**Table 2:** The association between NHHR and all-cause mortality among US cancer survivors 20 years of age and older, NHANES 1999–2018.

All-cause mortality	Crude model		Model 1		Model 2		Model 3	
	HR (95% CI)	*p*-value	HR (95% CI)	*p*-value	HR (95% CI)	*p*-value	HR (95% CI)	*p*-value
Pan-cancer								
**NHHR group**								
Higher NHHR (*n* = 3,546)	Ref		Ref		Ref		Ref	
Lower NHHR (*n* = 631)	1.23 (1.02–1.49)	0.029	1.29 (1.10–1.51)	0.002	1.32 (1.11–1.56)	0.001	1.31 (1.11,1.54)	0.001
Obesity-related cancers								
**NHHR group**								
Higher NHHR (*n* = 1,165)	Ref		Ref		Ref		Ref	
Lower NHHR (*n* = 240)	1.49 (1.14–1.94)	0.004	1.46 (1.12–1.90)	0.005	1.34 (1.01–1.77)	0.041	1.38 (1.06,1.80)	0.016

Crude model, no covariates were adjusted. Model 1, NHHR group, age, gender, race, education level, PIR and marital status were adjusted. Model 2, NHHR group, age, gender, race, education level, PIR, marital status, BMI, HEI2015, LTPA, smoking status, and alcohol status were adjusted. Model 3, NHHR group, age, gender, race, education level, PIR, marital status, BMI, HEI2015, LTPA, smoking status, alcohol status, diabetes, hypertension, cardiovascular disease, obesity-related tumors, and renal failure were adjusted. 95% CI, 95% confidence interval; HR, hazard ratio.

### Subgroup analysis of NHHR groups and all-cause mortality in cancer survivors

**Figure 4:**
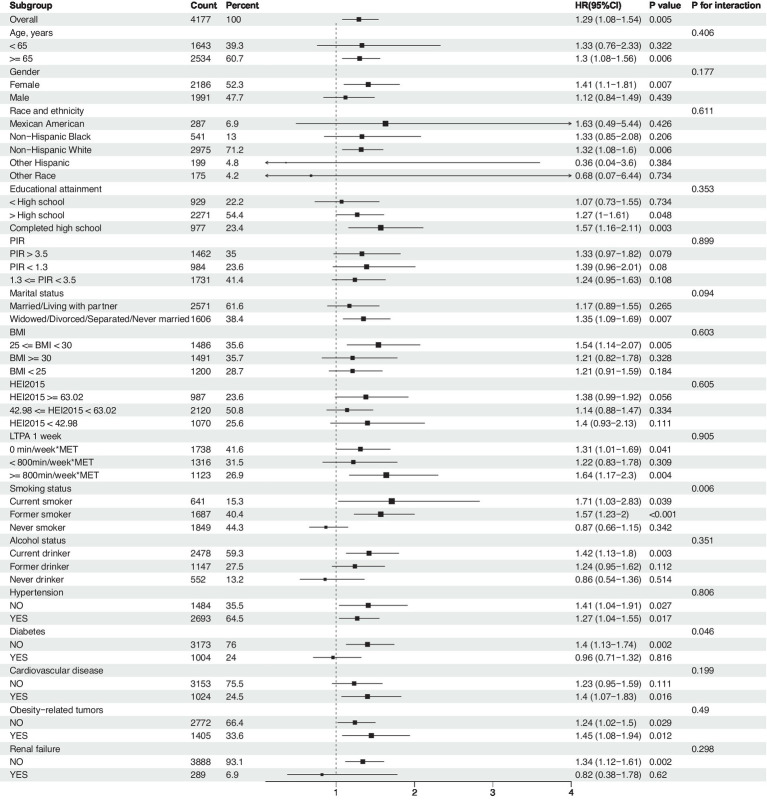
Association between NHHR and risk of all-cause mortality in cancer survivors, a subgroup analysis. Weighted cox proportional hazards models adjusted for age, gender, race, education level, PIR, marital status, BMI, HEI2015, LTPA, smoking status, alcohol status, diabetes, hypertension, cardiovascular disease, obesity-related tumors, and renal failure.

Subgroup analyses were conducted to assess the role of factors such as age, gender, race, education level, PIR, marital status, BMI, HEI-2015, LTPA, smoking and drinking status, hypertension, diabetes, cardiovascular disease, and renal failure in the association between NHHR and all-cause mortality. Results indicated that the association of NHHR with all-cause mortality remained consistent after adjustment for the aforementioned variables (Figure 4). Interaction effect analysis revealed that significant interactions with other factors were not observed (*P* interaction >0.05), with the exception of smoking status (*P* interaction = 0.006) and diabetes (*P* interaction = 0.046). Regarding smoking, current smokers demonstrated a higher risk of mortality (HR = 1.71, 95% CI: 1.03–2.83, *p* = 0.039), and former smokers also exhibited an increased risk (HR = 1.57, 95% CI: 1.23–2.00, *p* < 0.01), while the risk did not significantly increase for non-smokers (HR = 0.87, 95% CI: 0.66–1.15, *p* = 0.342). In terms of diabetes status, the all-cause mortality risk for cancer survivors with diabetes was not significantly associated with NHHR (HR = 0.96, 95% CI: 0.71–1.32, *p* = 0.816), whereas individuals without diabetes showed a significant association between NHHR and mortality risk (HR = 1.4, 95% CI: 1.13–1.74, *p* = 0.002). These findings suggest that smoking and diabetes status may be important moderators in the association between NHHR and all-cause mortality among cancer survivors.

### Non-linear association between NHHR groups and specific causes of death in cancer survivors

**Figure 5:**
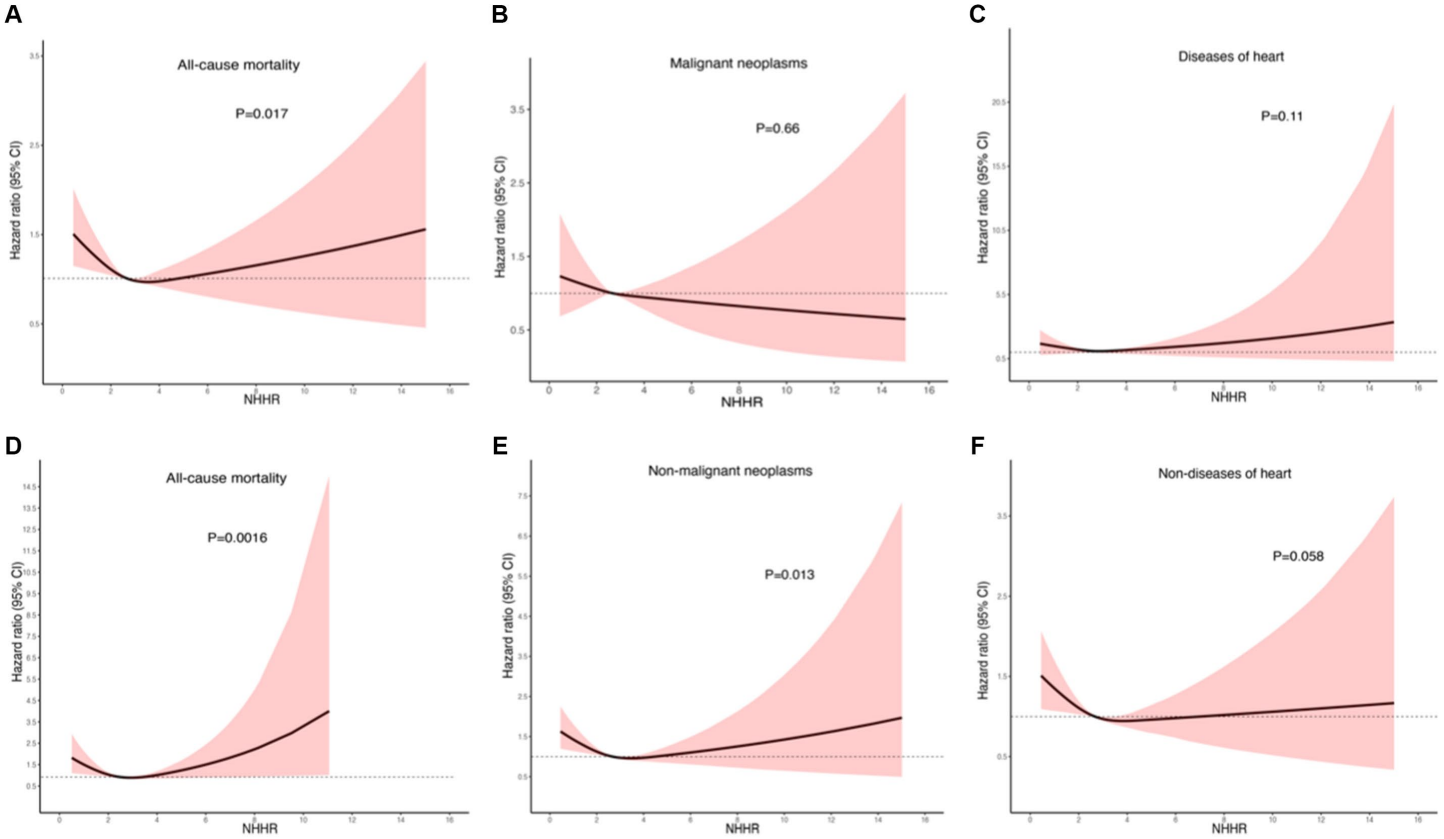
Nonlinear relationship between NHHR and mortality in adult cancer patients. **(A)** All-cause mortality; **(B)** cancer-specific mortality; **(C)** cardiac-specific mortality; **(D)** all-cause mortality in obesity-related tumors; **(E)** non-cancer-specific mortality; **(F)** non-cardiac-specific mortality. Statistical adjustments were made for age, gender, race, education level, PIR, marital status, BMI, HEI2015, PTLA, smoking status, alcohol status, diabetes, hypertension, cardiovascular disease, obesity-related tumors, and renal failure.

During the median follow-up period of 87 months, out of 4,177 cancer survivors, 1,458 individuals died, including 318 deaths from heart disease, 417 from cancer, and 723 from other causes. Through weight-adjusted multivariate Cox proportional hazards regression with restricted cubic splines (RCS), the following findings were observed: The effect of NHHR on all-cause mortality demonstrated a J-shaped relationship overall (Figure 5A), with a negative association when NHHR was below approximately 3.16 and a positive association when NHHR was above 3.16. The overall association test yielded a *p*-value of 0.024, and the test for non-linearity yielded a *p*-value of 0.017. The association between NHHR and cancer-specific mortality was not significantly inversely linear (Figure 5B), with an overall *p*-value of 0.48 and a non-linearity test *p*-value of 0.66. The analysis of the association between NHHR and cardiac-specific mortality revealed a J-shaped trend, although it did not reach statistical significance (Figure 5C), with an overall p-value of 0.27 and a non-linearity test *p*-value of 0.11. In the analysis of the association between NHHR and non-cancer-specific mortality, a similar J-shaped pattern with NHHR was observed (Figure 5E). The overall test for association produced a p-value of 0.01, and the test for non-linearity yielded a p-value of 0.013. The graphical analysis of NHHR in relation to non-cardiac-specific mortality (Figure 5F) indicated a negative correlation between the two, with an overall *p*-value of 0.031 and a non-linearity test *p*-value of 0.058. Notably, among patients with obesity-related cancers, the J-shaped association between NHHR and all-cause mortality was particularly significant (Figure 5D), with an overall *p*-value of 0.0068 and a non-linearity test *p*-value of 0.0016.

### Competing risk analysis of NHHR groups and specific causes of death in cancer survivors

**Figure 6:**
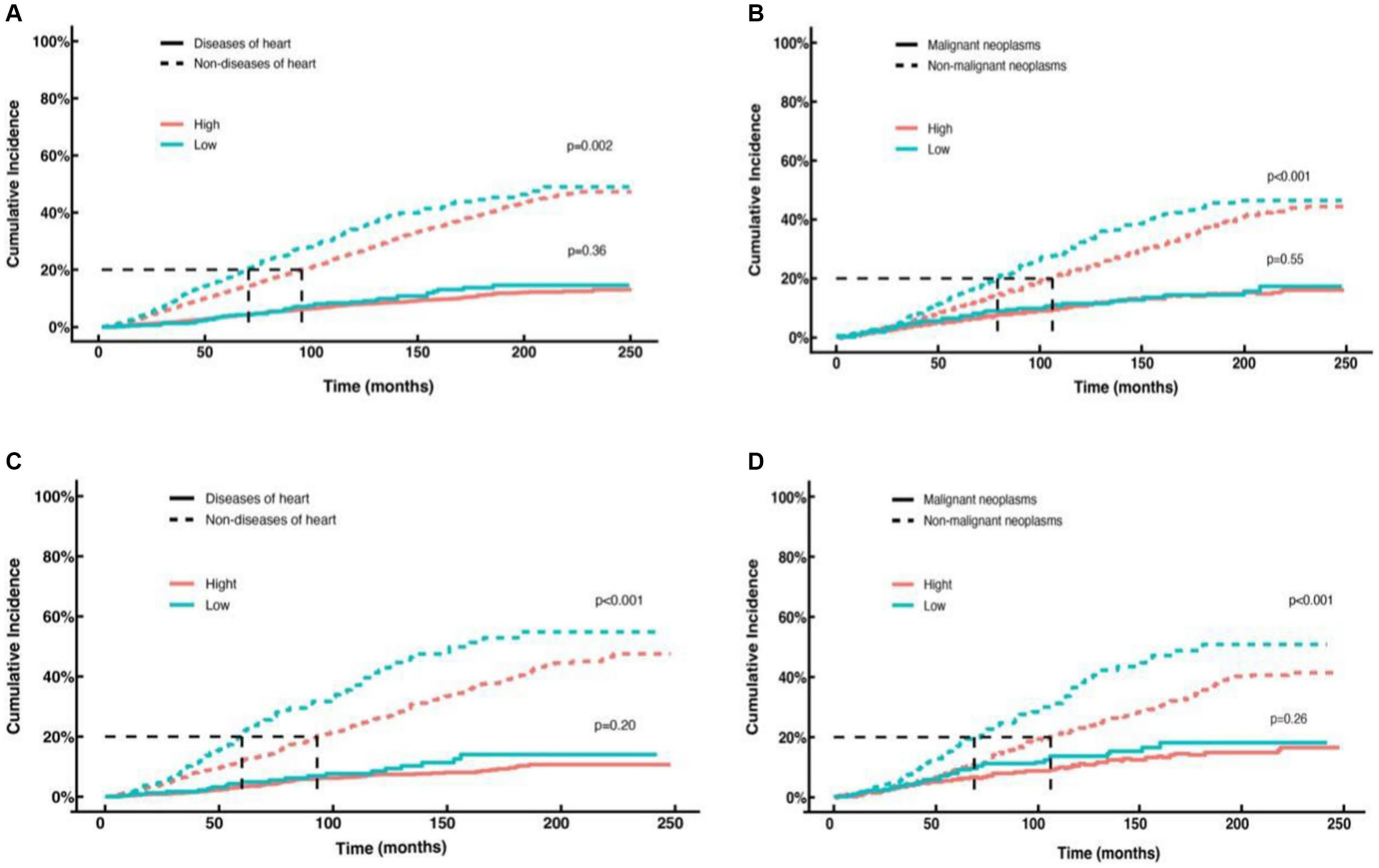
Competing risk curves for cardiac-specific and cancer-specific mortality in cancer survivors. **(A)** Cardiac-specific and non-cardiac-specific mortality in all cancer survivors in the high NHHR (red line) versus low NHHR (blue line) groups. **(B)** Cancer-specific and non-cancer-specific-specific mortality in all cancer survivors in the high NHHR (red line) versus low NHHR (blue line) groups. **(C)** Cardiac-specific and non-cardiac-specific mortality patients with obesity-related cancers in the high NHHR (red line) versus low NHHR (blue line) groups. **(D)** Cancer-specific and non-cancer-specific-specific mortality in patients with obesity-related cancers in the high NHHR (red line) versus low NHHR (blue line) groups.

In this study, the association between NHHR and specific causes of mortality, including cardiac and cancer-specific mortality, was examined across all cancer types as well as obesity-related cancers using a competing risks model. The key findings are as follows: In models for cardiac-specific mortality in pan-cancer survivors (Figure 6A) and obese cancer survivors (Figure 6C), no statistically significant difference in the risk of cardiac-specific mortality was observed between the low NHHR group and the high NHHR group after adjusting for competing events (*p* = 0.36 and *p* = 0.20, respectively). Nonetheless, the risk of non-cardiac-specific mortality was significantly higher in the low NHHR group compared to the high NHHR group after excluding cardiac-specific mortality (*p* = 0.002 and *p* < 0.001, respectively). In models for cancer-specific mortality in pan-cancer (Figure 6B) and obesity-related cancers (Figure 6D), no significant difference was found between the two groups in terms of cancer-specific mortality risk after controlling for competing events (*p* = 0.55 and *p* = 0.26, respectively). However, when excluding cancer-specific mortality, the risk of non-cancer-specific mortality was significantly higher in the low NHHR group compared to the high NHHR group in both models (both *p* < 0.001).

## Discussion

This investigation delves into the correlation between NHHR and all-cause as well as specific-cause mortality among cancer survivors, uncovering a heightened risk of all-cause mortality within the cohort with lower NHHR levels. A J-shaped curve was observed in relation to the association between NHHR and the all-cause mortality rate among cancer survivors, indicating an optimized NHHR range wherein mortality risks are minimized. Mortality risks are found to increase both above and below this optimal range, suggesting the crucial role of cholesterol management for the prognosis of survivors. The study identifies a nadir of all-cause mortality risk at an NHHR value of 3.16. In cause-specific mortality analyses, a negative correlation between NHHR and cancer-specific mortality and a J-shaped correlation with cardiac mortality were found. Though these trends may not be statistically significant, they do not preclude their potential biological and clinical relevance. Unaccounted variables or potential data quality issues may confound the interpretation of the results. It should also be noted that NHHR showed a potent correlation with other causes of death, such as those resulting from infections or cachexia-inducing illnesses, underscoring its potential as a biomarker in predicting a variety of mortality risks. Overall, this analysis provides significant biological insights into the role of NHHR in predicting mortality risk among cancer survivors and highlights the potential importance of managing NHHR levels for the long-term health maintenance of tumor survivors.

In discussing the differences between our research and similar studies, we first noticed that most prior research primarily focused on the association between HDL-C and mortality. For instance, one meta-analysis suggests an inverse correlation between HDL-C levels and all-cause and cardiovascular mortality (32). Another meta-analysis highlighted a relationship between higher TC and HDL-C levels and better cancer prognosis (27). Notably, some studies presented different results: a pooled analysis of 37 items in the general population (excluding high-risk groups, such as individuals with type 2 diabetes) demonstrated a J-shaped dose–response association between HDL-C and mortality from all causes, CVD, and cancer, and an L-shaped association with coronary heart disease mortality (28). Another study in a rural Chinese population suggested that both lower and higher HDL-C levels were associated with higher cardiovascular events rates, and lower HDL-C correlated with elevated cardiovascular mortality (33). Although these findings resonate with our results in the cancer population, our research goes a step further by including both non-HDL-C and HDL-C in the investigation, identifying the association between NHHR and all-cause and specific-cause mortality in cancer survivors, thus providing a more detailed perspective.

Contemporary studies focusing on lipid metabolism and cancer survival are scarce. A retrospective study in non-small cell lung cancer (NSCLC) indicated that patients with lower TC/HDL-C and non-HDL-C/HDL-C ratios had longer overall survival (OS), with the low non-HDL-C/HDL-C group experiencing a notably extended mean survival duration (59.00 vs. 52.35 months) (34). Our results slightly diverge from this study, potentially due to higher mortality rates associated with lung cancer and data heterogeneity. Another investigation of NSCLC patients undergoing radical resection and adjuvant chemotherapy showed that patients with HDL-C levels ≥1.52 mmol/L had a longer median DFS than those with low levels of NHHR (not reached vs. 26.30 months, *p* = 0.0005), whereas a decrease in HDL-C levels after chemotherapy was associated with a longer DFS (median DFS: 80.43 vs. 26.12 months, *p* = 0.0204). An increase in HDL-C levels by ≥0.32 mmol/L after chemotherapy was indicative of worse DFS (35). In women with obesity-associated cancers, high non-HDL-C levels above the 65th percentile correlated with increased risks of all-cause mortality (*p* = 0.01) and cardiovascular disease mortality (*p* = 0.003), but not with cancer-specific mortality (*p* = 0.37). Conversely, HDL-C levels above the 95th percentile were associated with lower rates of all-cause mortality (*p* = 0.002), and levels above the 65th percentile correlated with reduced cancer-specific mortality (*p* = 0.003), with no significant relationship to cardiovascular mortality (23).

Our research challenges the traditional belief that “higher levels of HDL-C are better.” Indeed, linear Mendelian randomization studies have demonstrated that genetic polymorphisms associated with an increase in HDL-C levels do not correlate with a reduced risk of cardiovascular events (36). Furthermore, pharmacological elevation of HDL-C does not translate to cardiovascular benefits (37). Critically, recent analyses have revealed that not only low but also high levels of HDL-C are associated with an increased risk of cardiovascular mortality (38).

Several hypotheses can be posited to explain these associations. HDL is a complex and heterogeneous family, consisting of subpopulations varying in size, density, shape, charge, and composition, which are subject to dynamic remodeling in circulation. Beyond its role as a cholesterol transporter, HDL exhibits multiple additional functionalities including cholesterol efflux capacity (CEC), antioxidative, anti-inflammatory, and immunomodulatory properties (39). Some individuals with HDL-C carry rare genetic variants that not only profoundly impact HDL-C levels but also lead to HDL dysfunctionality (40). Elevated levels of HDL-C may result from delayed catabolic metabolism (41). This could indicate an impairment in cholesterol transport to the liver for excretion. Similarly, prolonged residence in plasma could induce modifications in the compositional makeup of HDL particles, thus engendering functional impairment. The presence of larger-than-normal HDL particles at high HDL-C levels could be indicative of potential functional anomalies (41). Such large, potentially dysfunctional HDL particles could become entrapped within the arterial intima, contributing to cholesterol deposition and ultimately the pathogenesis of atherosclerosis and atherosclerotic cardiovascular disease (ASCVD).

Lipid metabolism plays a pivotal role in tumorigenesis and cancer progression. The state of low NHHR may reflect malnutrition and persistent inflammatory responses, which are closely linked to poor cancer prognoses (42, 43). Lipoproteins such as VLDL and LDL provide essential lipids and cholesterol for tumor cell growth, whereas HDL facilitate the removal of cholesterol from cancer cells, thereby affecting cellular homeostasis (44). Apolipoproteins and enzymes associated with HDL may exert antioxidative and anti-inflammatory effects, inhibit angiogenesis and apoptosis, and modulate immune responses, thus yielding anti-tumor effects (45). Dysregulated cholesterol metabolism, such as the accumulation of cholesterol in the tumor microenvironment, may directly contribute to tissue proliferation and tumor progression (46). To satisfy their increasing bioenergetic and therapeutic resistance needs, cancer cells undergo adaptive metabolic changes. This includes heightened uptake of LDL cholesterol and overexpression of LDL receptors across various cancer types, necessitating effective intracellular LDL processing and expedient distribution of LDL-derived cholesterol from the late endosome/lysosome system to organelles, facilitating tumor growth and dissemination (47). Recent findings underscore the cancer cells’ capacity to overcome nutrient scarcity by adapting metabolic pathways to catabolize alternative substrates such as proteins and lipids (48). This highlights the crucial role of lipid metabolism reprogramming in cancer, which not only fuels energy production and membrane biosynthesis but also mediates tumor development through lipid signaling pathways.

Hence, the functionality of HDL-C may be more critical than the levels of HDL-C. The multifunctional nature and complexity of HDL particles suggest that they may lose their protective roles across various disease states, potentially acquiring detrimental functionalities during conditions such as atherosclerosis, chronic diseases, or infections. It has been reported that the relationship between HDL-C levels and several diseases follows a U-shaped curve, where both low and extremely high levels of HDL-C are associated with increased risks of all-cause mortality, cardiovascular (CV) mortality, infections, diabetes, chronic kidney disease, autoimmune diseases, and dementia within the general population (26, 49, 50). Recent studies have demonstrated that measurements of various potential HDL functionalities, such as CEC and the HDL inflammatory index (i.e., the capacity of HDL to inhibit LDL oxidation) or the circulating quantity of HDL particles, serve as better predictors for CV events than merely the cholesterol content of HDL (51–53).

While this study provides preliminary evidence of the prognostic marker role of NHHR in cancer survivors, its limitations warrant consideration. Firstly, despite attempts to control for confounding factors through multivariable adjustment, residual bias from unmeasured variables cannot be entirely excluded. This is particularly pertinent given that the tumor data from the NHANES database used largely rely on self-reporting by participants, thereby raising the possibility of diagnostic bias. Additionally, due to NHANES encompassing pan-cancer data and the limited sample size for each cancer type without detailed subtyping, staging, and treatment modalities, the generalizability of the study findings is constrained. In analyses specific to causes of death, it is important to recognize the heterogeneity in the causes of death attributed to cancer, where cardiovascular-specific mortality may exhibit a U-shaped relationship, whereas coronary heart disease-related deaths may display a different pattern. These complexities suggest the need for more comprehensive and standardized longitudinal data for in-depth analyses in future research. Furthermore, the association of HDL levels with daily life habits such as diet and exercise suggest that examining the transient state of NHHR might not accurately assess its association with long-term mortality risk. Future studies should implement extended follow-ups and repeated measures of NHHR for a better understanding of its level fluctuations and long-term impact on the prognosis of cancer survivors. Our analysis is also limited by the statistical methods used, which did not determine the optimal NHHR cutoff values for survival analysis with complex sampling data. Moreover, given the scarcity of individuals with an NHHR value greater than 8, our model was ineffective in predicting survival outcomes for this subgroup of cancer survivors. Lastly, as an observational study, the conclusions drawn cannot directly infer a causal relationship between NHHR levels and mortality risk among cancer survivors.

Future studies should employ large-scale and multi-center datasets to further validate the applicability and efficacy of NHHR as a predictive marker in clinical practice and to examine its consistency across different populations and types of cancer. Simultaneously, exploring the specific biological mechanisms underlying the association between NHHR and all-cause mortality as well as disease-specific mortality among cancer survivors will enhance our understanding of its predictive value and guide future intervention strategies. Moreover, as our comprehension of the tumor microenvironment and the metabolic state of cancer survivors deepens, clearer guidance on the clinical application of NHHR may emerge. We will further measure NHHR values in both cancerous and non-cancerous populations, adjusting for the use of lipid-lowering medication, atherosclerosis, metabolic syndrome, and other confounding factors, to analyze the distribution of lipids in cancer populations and their impact on cardiovascular diseases.

## Conclusion

In the domain of lipid metabolism and its correlation with the prognosis of cancer survivors, the findings of this study offer novel perspectives and foundational information vital for future clinical application and biomarker research. Subsequent investigations may build upon this work to further explore the interplay between NHHR and specific cancer types, diverse treatment regimens, and lifestyle interventions, with the aim of identifying more instructive preventative and therapeutic strategies.

## Data Availability

Publicly available datasets were analyzed in this study. This data can be found here: National Health and Nutrition Examination Survey (https://www.cdc.gov/nchs/nhanes/index.htm).

## Footnotes

^1^ https://www.cdc.gov/

^2^ https://CRAN.R-project.org/package=maxstat

## Glossary

CVD: Cardiovascular disease
PLC: Plasma lipoprotein cholesterol
HDL-C: High-density lipoprotein cholesterol
non-HDL-C: Non-HDL cholesterol
LDL-C: Low-density lipoprotein cholesterol
NHANES: National Health and Nutrition Examination Survey
NHHR: Non-HDL-C/HDL-C ratio
NCHS: National Center for Health Statistics
CDC: Centers for Disease Control and Prevention
MAR: Missing at Random
SD: Standard Deviation
RCS: Restricted Cubic Spline
ROC: Receiver Operating Characteristic
AUC: Area Under the Curve
HR: Hazard Ratio
CI: Confidence Interval
OS: Overall survival
CEC: Cholesterol efflux capacity
ASCVD: Atherosclerotic cardiovascular disease
PIR: Poverty Income Ratio
BMI: Body Mass Index
HEI2015: Healthy Eating Index-2015
LTPA: Leisure time physical activity
STROBE: Strengthening the Reporting of Observational Studies in Epidemiology
